# Primary intracranial malignant melanoma in a pediatric patient: A rare case report

**DOI:** 10.1016/j.jdcr.2025.07.004

**Published:** 2025-07-25

**Authors:** Annabelle Huntsman, Laura Mims, Shadai Gociman, Casey J. Mehrhoff

**Affiliations:** a School of Medicine, University of Utah, Salt Lake City, Utah; b Division of Pediatric Hematology-Oncology, Primary Children’s Hospital, University of Utah, Salt Lake City, Utah; c Department of Dermatology, University of Utah, Salt Lake City, Utah

**Keywords:** melanocytic neoplasm, pediatric dermatology, pediatric melanoma, primary CNS melanoma, primary intracranial malignant melanoma (PIMM)

## Introduction

Melanoma incidence in adults has historically correlated with increasing age and UV exposure.^1^ In the pediatric population, however, melanoma is relatively rare with just 2% of all new cancer-related cases in patients aged <20 years.^2^ Primary central nervous system (CNS) melanoma, the rarest subtype of melanocytic neoplasm, makes up just 1% of all melanomas across all age groups and typically exhibits aggressive behavior.^3^

Among primary CNS melanomas, primary intracranial malignant melanoma (PIMM) is an exceedingly uncommon variant arising from leptomeninges characterized by neoplastic growth of melanocytes within the CNS.^4^^,^^5^ Microscopically, PIMM often exhibits characteristics of melanocytic differentiation that closely resemble cutaneous melanoma, making differentiating PIMM from a metastatic CNS lesion a challenging task.

To date, very few cases of PIMM have been reported in the pediatric population. This exceedingly small sample size and lack of protocolized standard of care have likely contributed to the poor overall survival (9-25 months) in children diagnosed with PIMM.^6^ Here, we present a case of a pediatric patient with PIMM who presented with a seizure and a growing lesion located in the posterior aspect of the right Sylvian fissure.

## Case report

An 8-year-old boy presented to the hospital with multiple seizures. A brain magnetic resonance imaging (MRI) revealed a 5 mm lesion in the posterior aspect of the right Sylvian fissure/inferior operculum appearing consistent with characteristics concerning for a small, cavernous malformation. Neurosurgery recommended a repeat brain MRI in 3 months, but he was lost to follow-up.

Behavioral concerns progressed 5 months after the initial presentation, leading to readmission. Repeat brain MRI revealed the brain lesion had grown to 6 × 10 mm.

**Fig 1:**
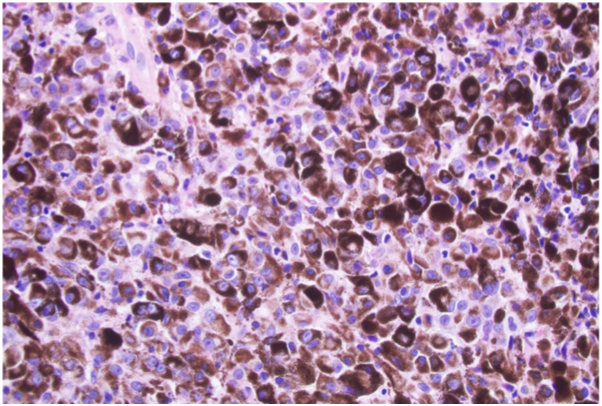
Gross brain tumor specimen (0.5 × 2 cm) demonstrating black pigmentation consistent with melanoma.

**Fig 2:**
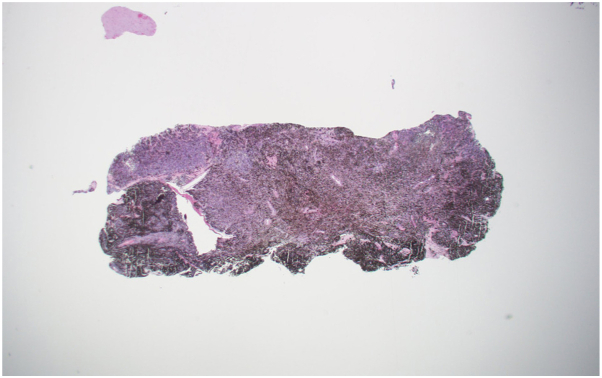
Microscopic brain tumor sample showing a pigmented discoloration and stained positive for tumor markers Melan-A and HMB45.

The patient underwent a gross total resection to remove the brain lesion, at which time neurosurgery noted that the specimen appeared black, which was also appreciated on low-power microscopic inspection of the histologic specimen (Fig 1). The pathologic examination also highlighted that this lesion was Melan-A and HMB45 positive (Fig 2), consistent with a PIMM. Dermatology was consulted for concern of possible neurocutaneous melanosis due to an approximately 3 × 4 cm light brown well-demarcated macule noted on the patient’s right arm, consistent with a café au lait macule. No skin lesions suggested primary cutaneous melanoma. Ophthalmology ruled out a primary uveal melanoma with brain metastases, with no evidence of uveal melanoma, therefore confirming the diagnosis of primary CNS melanoma. Cerebrospinal fluid analysis revealed no tumor cells were present and positron emission tomography scan confirmed no sites of metastasis. The patient received 30 Gy of proton radiation to the brain lesion, with follow-up brain MRIs showing no residual disease.

Patient’s course to date has been complicated by persistent behavioral concerns, leading to repeat psychiatric admissions. However, follow-up brain and spine MRIs, now 15 months since completing radiation treatment, have been reassuring with no recurrent disease. He will continue to follow up regularly with oncology, dermatology, and ophthalmology.

## Discussion

PIMM is a rare and highly aggressive malignant diagnosis in the pediatric population. Only 12 other case reports are known in the literature^5^^,^^6^; making our patient the 13th reported pediatric case, and the youngest reported case at 8 years old. Most pediatric patients with PIMM are adolescents (ages 11-17 years). Overall survival for those patients has been reported to be aged between 9 and 24 months. Our patient falls within this timeframe, as he is 16 months postdiagnosed and will continue with close interval follow-up.

The first-line treatment for PIMM is gross total resection, resulting in significantly longer overall survival time than subtotal resection or a biopsy.^6^ However, there are minimal data on the role of adjuvant therapy such as chemotherapy, immunotherapy, and/or irradiation. Previous case reports report irradiation has been used more as a palliative treatment option rather than as an upfront therapy in pediatric patients,^7^ and melanoma historically has been insensitive to standard doses of irradiation.^7^

However, in an adult literature review from China, Chen et al^7^ report that studies have found neoadjuvant radiation therapy after surgery has significantly reduced the risk of local recurrence compared with total gross resection alone, although the overall prognosis remains poor. Our patient had a gross total resection followed by 30 Gy of proton irradiation and has remained disease-free since that time. However, we should not discount the toxicity of radiation therapy especially in pediatric patients.

Moreover, NRAS a common genetic driver of PIMM has been studied in mouse models and shown to be sensitive to MEK inhibitors. Thus, MEK inhibitors could be a potential treatment for these patients. The mitogen-activated protein kinase pathway is a cellular process that helps control cell growth and survival. Mutations in NRAS can activate this pathway, leading to increased cell growth and, ultimately, malignancy. Although preclinical data have been promising, no clinical trial has yet shown MEK inhibitors to be effective against tumors with NRAS mutations.^6^

Our patient’s molecular testing of the tumor was positive for BAP1p.R385 and GNAQp.R183 (pathogenic), as well as monosomy 3 and isochromosome 8q (likely pathogenic). GNAQ gene mutations are most commonly seen in uveal melanomas (80%-90% of cases^8^). Similar to NRAS, these GNAQ gene mutations have shown sensitivity to MEK inhibitors. There are downstream signaling pathways through the mitogen-activated protein kinase pathway that regulate the expression of proliferative signals. However, several clinical trials have investigated monotherapy selumetinib (MEK inhibitor) compared with chemotherapy in uveal melanoma with no significant change in overall survival.^8^ Because there are limited data on the effectiveness of MEK inhibitors for PIMM and because the patient was treated with surgical resection and radiation, we chose not to use a MEK inhibitor as first-line treatment.

This case report emphasizes the rarity of PIMM in pediatric patients, especially those aged <11 years, and the limited data available on effective treatments for PIMM. Gross total resection with or without radiation therapy has the best overall survival, although the prognosis remains poor. Moreover, this report has broader implications on the field of dermatology, as a key part of the multidisciplinary care management of these patients with regular total body skin examinations and frequent monitoring.

## Conflicts of interest

None disclosed.
